# The Interplay Between Oophorectomy and Alzheimer’s Disease: A Narrative Review

**DOI:** 10.3390/medicina62102043

**Published:** 2026-10-21

**Authors:** Shuiqing Xu, Xiaotong Fu, Lijun Zuo, Ming Wang

**Affiliations:** 1 Department of Gynecologic Oncology, Beijing Obstetrics and Gynecology Hospital, Capital Medical University, Beijing Maternal and Child Health Care Hospital, Beijing 100006, China; xsq@mail.ccmu.edu.cn; 2 Department of Neurosurgery, Beijing Chaoyang Hospital, Capital Medical University, Beijing 100006, China; fuxiaotong98@163.com; 3 Department of Neurology, Beijing Tiantan Hospital, Capital Medical University, Beijing 100006, China

**Keywords:** Alzheimer’s disease, oophorectomy, surgical menopause, estrogen, cognitive decline, dementia, hormone replacement therapy

## Abstract

Alzheimer’s disease (AD) is the leading cause of dementia worldwide and affects women more frequently than men. Although age remains the main risk factor, reproductive aging and changes in ovarian hormone exposure may also contribute to this difference. Premenopausal bilateral oophorectomy causes an abrupt loss of ovarian hormones, including estradiol, progesterone, and testosterone, and several observational studies have associated this change with a higher risk of later cognitive impairment or dementia, particularly when surgery is performed at a younger age. However, these studies have assessed different outcomes, including dementia, mild cognitive impairment, subjective cognitive changes, and AD-related imaging findings, and current evidence does not establish a direct causal relationship between oophorectomy and AD. Possible biological pathways include neuroinflammation, synaptic dysfunction, amyloid-β and tau-related changes, and cerebrovascular dysfunction, although much of the mechanistic evidence remains experimental or indirect. Genetic background, vascular health, age at surgery, and the duration of hormone deprivation may further influence individual risk. Hormone replacement therapy has an established role in the management of premature or early surgical menopause when there is no contraindication, but its effect on long-term AD prevention remains uncertain; the timing of treatment may be important, but current evidence does not confirm a preventive effect. This review summarizes current epidemiological, neuroimaging, and experimental evidence linking oophorectomy with cognitive health and AD, and discusses hormone therapy, pharmacological and non-pharmacological management, emerging research approaches, and remaining knowledge gaps. Future studies with detailed information on surgical timing, hormone exposure, genetic risk, vascular factors, longitudinal cognition, and AD-related biomarkers are needed to better define which women may be most vulnerable after premenopausal oophorectomy.

## 1. Introduction

Alzheimer’s disease (AD) is the most common cause of dementia worldwide. Dementia affected an estimated 57.4 million people globally in 2019, and this number is expected to increase to approximately 152.8 million by 2050 [1]. Women’s longer life expectancy has traditionally been considered an important reason for this difference; however, longer survival alone may not fully explain the higher burden of AD in women. Biological changes that are specific to women, particularly reproductive aging and the decline in ovarian hormones during menopause, may also be associated with susceptibility to AD [2,3].

AD is a multifactorial disease, and its development is influenced by both genetic background and environmental or lifestyle factors. The apolipoprotein E (APOE) ε4 shows a strong gene-dose effect, particularly in homozygotes, with the risk increasing by approximately 2–3-fold in heterozygous carriers and 10–15-fold in homozygous carriers [4]. Other genetic and lifestyle factors may also affect dementia risk, but they are not the main focus of this review. Since this review mainly discusses oophorectomy and ovarian hormone loss, greater attention is given to reproductive aging and the changes that occur after surgical menopause. Unlike natural menopause, in which ovarian hormone levels usually decline over several years, bilateral oophorectomy causes a much more sudden loss of ovarian hormones, and this difference may be important when considering later cognitive changes.

Against this general background of genetic, environmental, and lifestyle factors, reproductive factors have also received increasing attention. Oophorectomy refers to the surgical removal of one or both ovaries and may be performed for several reasons, including treatment of benign ovarian conditions such as endometriosis or ovarian cysts, reduction in cancer risk in women with hereditary cancer syndromes such as BRCA1/2 pathogenic variants, and, in some cases, as part of gender-affirming surgery [5,6]. Bilateral salpingo-oophorectomy (BSO) is an important strategy for reducing ovarian cancer risk in BRCA1/2 mutation carriers, who face lifetime ovarian cancer risks of approximately 40% (BRCA1) and 18% (BRCA2) [7]. For this reason, risk-reducing surgery is generally recommended at a relatively young age, commonly around 35–40 years for BRCA1 carriers and 40–45 years for BRCA2 carriers [8]. When BSO is performed before natural menopause, hormone replacement therapy (HRT) may be considered to relieve menopausal symptoms and reduce some of the longer-term consequences associated with premature loss of ovarian function [8].

The consequences of oophorectomy, however, are not limited to cancer prevention and menopausal symptoms. When both ovaries are removed before natural menopause, circulating ovarian hormones fall rapidly rather than gradually, which is different from the slower hormonal change seen during natural menopause and may lead to different effects on the brain and other hormone-sensitive systems. A 2024 systematic review and meta-analysis by Hassan et al. reported that hysterectomy with BSO in women younger than 50 years was associated with an increased risk of dementia (HR = 1.70, 95% CI = 1.07–2.69), coronary heart disease (HR = 1.17, 95% CI = 1.10–1.25), stroke (HR = 1.20, 95% CI = 1.10–1.31), and depression (HR = 1.39, 95% CI = 1.22–1.60), while a lower risk of breast cancer was observed (HR = 0.78, 95% CI = 0.73–0.84) [9]. Similar findings have been reported for cognitive outcomes; Georgakis et al. reported in a meta-analysis of 11 studies (N = 18,867) that early surgical menopause (≤45 years) was associated with a 70% increased risk of dementia (HR = 1.70, 95% CI = 1.07–2.69) and changes in verbal memory, semantic memory, and processing speed [10]. The same risk estimate was reported in these two meta-analyses, but the available evidence is partly overlapping, so these findings should not be viewed as two completely independent estimates. Although the findings are not completely consistent across all studies, the available evidence suggests that loss of ovarian function at a younger age may be related to later cognitive decline and therefore deserves further attention.

Based on these observations, this review focuses on the possible relationship between oophorectomy, loss of ovarian hormones, and AD. We first summarize epidemiological studies examining cognitive decline and dementia after oophorectomy, with particular attention to age at surgery and the timing of surgical menopause; we then discuss several possible biological pathways, including neuroinflammation, synaptic dysfunction, amyloid-β and tau-related changes, and cerebrovascular injury. Hormonal treatment and other pharmacological or non-pharmacological approaches are also considered, together with newer therapeutic strategies and areas where current evidence remains limited. Because the available studies do not all use the same outcome, this review distinguishes clinically diagnosed AD from all-cause dementia, mild cognitive impairment, subjective cognitive changes, and imaging or biomarker findings whenever possible. By bringing together findings from clinical cohorts, neuroimaging studies, preclinical models, and genetic research, this review aims to provide a clearer overview of what is currently known about ovarian hormone loss and cognitive health after oophorectomy, while also identifying questions that still need further study.

## 2. Methods

This review was written as a narrative review, with reference to the Scale for the Assessment of Narrative Review Articles (SANRA) to improve the transparency of the literature selection and reporting process [11]. The review mainly focused on premenopausal oophorectomy, surgical menopause, cognitive changes, dementia, and Alzheimer’s disease. Related evidence on hormone therapy, neuroimaging, and possible biological mechanisms was also considered.

Relevant studies were identified through searches of PubMed/MEDLINE, Web of Science, and Scopus, from database inception to 9 October 2026. Search terms included combinations of “oophorectomy”, “bilateral oophorectomy”, “bilateral salpingo-oophorectomy”, “surgical menopause”, “premature menopause”, “Alzheimer’s disease”, “dementia”, “cognitive impairment”, “mild cognitive impairment”, “estrogen”, “estradiol”, “hormone replacement therapy”, and “menopausal hormone therapy”. Additional terms related to neuroinflammation, amyloid-β, tau, cerebrovascular changes, and estrogen receptors were used for the mechanistic sections. Reference lists of relevant reviews and key original studies were also checked.

Priority was given to cohort studies, prospective studies, clinical trials, systematic reviews, and meta-analyses directly related to oophorectomy, surgical menopause, hormone therapy, or cognitive outcomes. Neuroimaging and biomarker studies were included when relevant. Animal and cellular studies were mainly used to discuss possible mechanisms.

Because this was a narrative review, no formal meta-analysis or GRADE assessment was performed. The evidence was evaluated qualitatively according to study design, directness to the review question, outcome definition, follow-up duration, adjustment for confounding, and major risk of bias. Differences in age at surgery, indication for oophorectomy, hysterectomy status, hormone therapy use, and outcome type were considered during interpretation. Studies were selected according to their direct relevance to the review question, with preference given to original human studies and higher-level evidence where available.

## 3. Clinical and Epidemiological Evidence Linking Oophorectomy to Cognitive Decline and Dementia

### 3.1. Impact of Surgical Menopause on Cognitive Decline

Surgical menopause induced by bilateral oophorectomy has been associated in several studies with a higher risk of later cognitive impairment and dementia, although the reported strength of this association varies and appears to be influenced by age at surgery. One of the earlier reports from the Mayo Clinic Cohort Study of Oophorectomy and Aging showed that premenopausal oophorectomy was associated with a higher long-term risk of cognitive impairment or dementia, and the association was stronger when surgery was performed at a younger age [12]. These findings were important because they first suggested that the timing of ovarian hormone loss may influence later cognitive health. Later analyses from the same cohort continued to show an age-related pattern, although the outcomes assessed were mainly cognitive impairment and mild cognitive impairment rather than clinically diagnosed AD.

The Mayo Clinic Cohort Study of Oophorectomy and Aging provides important long-term evidence in this area; women who underwent premenopausal bilateral oophorectomy before age 46 years had more than twice the odds of developing mild cognitive impairment (MCI) compared to women with intact ovaries (aOR = 2.21, 95% CI = 1.41–3.45, *p* < 0.001), and cognitive assessment was performed a median of approximately 30 years after surgery [13]. This association was stronger among women whose oophorectomy was performed for benign ovarian conditions (aOR = 2.43, 95% CI = 1.36–4.33) rather than for cancer prophylaxis, while the study did not show a clear reduction in this association among women who received estrogen therapy after surgery [13]. These results should therefore be interpreted as evidence for later cognitive impairment rather than direct evidence of AD. The Danish Nurse Cohort study, which prospectively included 24,851 women, reported an 18% higher rate of dementia following bilateral oophorectomy (aRR = 1.18, 95% CI = 0.89–1.56); however, the confidence interval included 1.0 and the association did not reach statistical significance. The same study also found that unilateral oophorectomy was associated with a non-significant 13% lower dementia rate, and neither hysterectomy nor HRT use significantly modified the association [14]. These findings suggest that the association is not uniform across different cohorts and types of surgery. Data from the Rush cohort also support a possible age-related effect. In that study, younger age at surgical menopause was associated with faster decline in several cognitive domains and with a greater burden of AD-related neuropathological changes later in life [15]. However, this was still an observational study, and age at surgery, the reason for surgery, hormone exposure, and other health factors may have influenced the findings.

Additional neuroimaging evidence points in a similar direction, although these studies measured brain structure or subjective cognitive symptoms rather than clinical AD. A Canadian cohort study found that women with early bilateral oophorectomy had lower gray matter volume in estradiol-sensitive brain regions, together with approximately twice the odds of subjective cognitive decline (SCD) at an average of 5 years post-surgery [16]. Neuroimaging studies have also reported structural and biomarker differences many years after premenopausal oophorectomy. Kantarci et al. reported that early premenopausal bilateral oophorectomy was associated with thinner entorhinal cortex, higher Aβ load, higher tau load at higher Aβ levels, and greater AD-related cortical atrophy later in life [17]. Similarly, Mielke et al. found that women who underwent PBO before age 40 years had lower white matter integrity, reflected by lower fractional anisotropy (FA) and higher mean diffusivity (MD), across multiple brain regions, including the anterior corona radiata, genu of the corpus callosum, and inferior fronto-occipital fasciculus [18]. Estrogen replacement did not clearly attenuate these white matter differences; however, the observational design does not establish a causal effect of early hormone loss on later-life white matter integrity [18].

Changes after surgical menopause may also differ across individual cognitive domains rather than affecting all aspects of cognition in the same way. The prospective WHAM (What Happens After Menopause) study followed premenopausal BRCA1/2 carriers undergoing risk-reducing BSO for 24 months and found that most cognitive changes over time were similar to those in the comparison group, while verbal learning showed a small but statistically significant lower improvement after surgery [19]. Among women who underwent RRSO, verbal learning performance was also better in HRT users than in non-users, although this finding does not prove that HRT prevents cognitive decline. These results suggest that the short-term cognitive effect of RRSO may be relatively small and may not affect all cognitive domains in the same way. A smaller prospective study also found a significant change in self-reported cognitive symptoms at 6 months after RRSO, while most other cognitive domains remained relatively stable [20]. Because this study had a short follow-up and relied mainly on subjective cognitive measures, the findings are difficult to interpret as evidence of either improvement or deterioration in long-term cognition. Changes in anxiety or concern about ovarian cancer after surgery may also have influenced self-reported cognitive symptoms. Taken together, these short-term findings appear more modest than the associations reported in studies with follow-up extending into later life.

Genetic factors, particularly APOE ε4 carrier status, may further influence the relationship between early oophorectomy and later cognitive outcomes. The UK Biobank study by Calvo et al. reported that women carrying APOE ε4 who underwent early BSO had a greater burden of AD-related risk factors and appeared to show greater cognitive vulnerability [21]. However, the available data do not establish a definite interaction between APOE ε4 and surgical menopause, and the findings should not be interpreted as showing that APOE ε4 specifically explains the cognitive changes after oophorectomy. Therefore, APOE ε4 may be considered a possible modifying factor at present, rather than a confirmed explanation for the cognitive changes observed after early oophorectomy.

### 3.2. Hormonal Changes Following Oophorectomy

Bilateral oophorectomy causes a rapid and marked decline in circulating ovarian hormones, including 17β-estradiol, progesterone, and testosterone, and this endocrine change leads to surgical menopause. In premenopausal women, the hormonal decline occurs much more abruptly than during natural menopause, in which ovarian hormone levels usually change gradually across the several-year menopausal transition [22]. This difference in the speed of hormone loss is important, because the brain and other hormone-sensitive tissues may have less time to adapt after surgery than during natural menopause. Among these hormones, estradiol has received the most attention because estrogen signaling is involved in several processes related to brain function; 17β-estradiol can influence synaptic plasticity through N-methyl-D-aspartate (NMDA) receptor activity, support neurogenesis in the hippocampal dentate gyrus, participate in the regulation of cerebral blood flow through endothelial nitric oxide synthase (eNOS), and reduce inflammatory responses by affecting microglial activity [23,24]. Beyond estradiol, oophorectomy also reduces ovarian androgen production and alters the hypothalamic-pituitary-ovarian (HPO) axis; after the loss of ovarian negative feedback, follicle-stimulating hormone (FSH) and luteinizing hormone (LH) levels rise [25]. FSH has also attracted attention in relation to AD. Experimental studies suggest that increased FSH signaling may contribute to amyloid-β production and tau phosphorylation, but most of this evidence comes from animal or experimental models, and direct evidence in women after oophorectomy is still limited [26]. Therefore, increased FSH should currently be considered a possible related pathway rather than a confirmed cause of cognitive decline after surgical menopause. Furthermore, hysterectomy alone—even without oophorectomy—may affect hormonal homeostasis by altering utero-ovarian blood flow; this may reduce ovarian perfusion and has been associated with an earlier onset of natural menopause in some studies [27].

Human neuroimaging studies have also reported brain structural differences in women with a history of early bilateral oophorectomy, although these observational findings cannot determine whether the changes are caused directly by hormone loss. Later-life imaging studies have reported reduced entorhinal cortex thickness, greater amyloid-β burden, and other AD-related structural changes in women who underwent premenopausal bilateral oophorectomy [17], while other studies have shown lower white matter integrity in women who underwent surgery at a younger age [18]. These findings suggest that early ovarian hormone loss may be associated with long-term brain changes, but they should not be interpreted as direct evidence that oophorectomy causes AD. In the UK Biobank neuroimaging substudy, HRT users with a history of hysterectomy plus BSO showed a slightly younger estimated brain age on gray matter brain age gap (GM-BAG) measures (mean difference = 0.77 years) compared with HRT users without such surgical history [28]. This result suggests that hormone therapy use may be related to differences in some brain-aging measures, but the study was observational and was not designed to prove a protective effect of HRT on cognition or AD risk. The findings also should not be extended to other outcomes, such as cerebral perfusion or white matter injury, unless those outcomes were directly assessed. The main clinical and neuroimaging studies examining cognitive and brain outcomes after oophorectomy are summarized in Table 1.

### 3.3. Influence of Oophorectomy Timing on AD Risk

The timing of oophorectomy relative to natural menopause appears to be an important factor associated with later cognitive risk, and this age-related pattern is also relevant to the “critical window” or “window of opportunity” hypothesis. According to this hypothesis, the effects of ovarian hormones and exogenous hormone therapy on the brain may depend partly on the stage of reproductive aging at which hormone loss or treatment occurs; responsiveness to estrogen may gradually change as women move through menopause and later aging [29]. However, this hypothesis is mainly supported by observational and experimental evidence, and it has not been confirmed as a clear preventive strategy for AD in women after oophorectomy. Epidemiological findings provide some support for an age-related difference. The Georgakis meta-analysis found that surgical menopause at any age was not significantly associated with dementia risk (HR = 1.16, 95% CI = 0.96–1.43), whereas early surgical menopause (≤45 years) was associated with a higher risk (HR = 1.70, 95% CI = 1.07–2.69) [10]. These findings suggest that the age at which ovarian function is lost may be more important than the presence of surgical menopause alone. The 2021 update by Rocca et al. also described an age-related pattern, with the association between bilateral oophorectomy and cognitive impairment appearing stronger when surgery was performed at a younger age. Women who underwent bilateral oophorectomy before age 40 appeared to have the greatest risk, while the reported association became weaker among women undergoing surgery at ages 40–45 and 46–49 years [30]. Taken together, these findings suggest that earlier loss of ovarian function may be related to greater later cognitive vulnerability, but the results are still based mainly on observational studies and may be influenced by the reason for surgery, baseline health, hormone therapy use, and other factors.

For women with BRCA1/2 pathogenic variants, however, age at surgery cannot be considered only from the perspective of cognitive health; the timing of risk-reducing salpingo-oophorectomy (RRSO) also needs to balance cancer prevention against the possible long-term consequences of premature surgical menopause, including cardiovascular disease, bone loss, and cognitive decline [8,30]. Risk-reducing surgery is therefore recommended at a relatively young age for many BRCA1/2 carriers, commonly around 35–40 years for BRCA1 and 40–45 years for BRCA2 carriers; hormone therapy may also be considered after premenopausal surgery when there is no contraindication [8]. This balance is important because delaying surgery may reduce the duration of hormone loss but may also increase cancer-related risk in women who carry high-risk pathogenic variants.

One possible explanation for the effect of timing is that brain responsiveness to ovarian hormones changes with age and with the duration of hormone deprivation. Before menopause, estrogen signaling is involved in synaptic plasticity, neurogenesis, and several processes related to learning and memory; as reproductive aging progresses, changes in synaptic function, vascular health, and estrogen receptor signaling may alter the response to both endogenous and externally administered estrogen [3,29]. Changes in estrogen receptor expression, including ERα in brain regions related to cognition, have also been proposed as part of this process, although the changes are unlikely to be explained by a single receptor pathway alone [3]. Animal studies provide additional support for a timing-dependent response; in ovariectomized rodents, estradiol given relatively soon after ovariectomy has preserved some cholinergic and memory-related measures, whereas treatment after a longer period of hormone deprivation has generally shown less benefit [31]. These experimental findings support the biological plausibility of the critical-window hypothesis, but they should not be interpreted as direct evidence that the same treatment timing prevents AD in humans. Differences between rodent ovariectomy models and human surgical menopause, including age, hormone exposure, and duration of follow-up, also limit direct comparison.

The long-term cognitive effects of oophorectomy in transgender populations remain unclear. Because the available studies in this area mainly describe endocrine or ovarian changes rather than cognitive outcomes, this topic is discussed later as an important gap for future research rather than as current evidence for AD risk.

## 4. Pathophysiological Mechanisms Linking Hormone Loss to Alzheimer’s Disease

**Figure 1:**
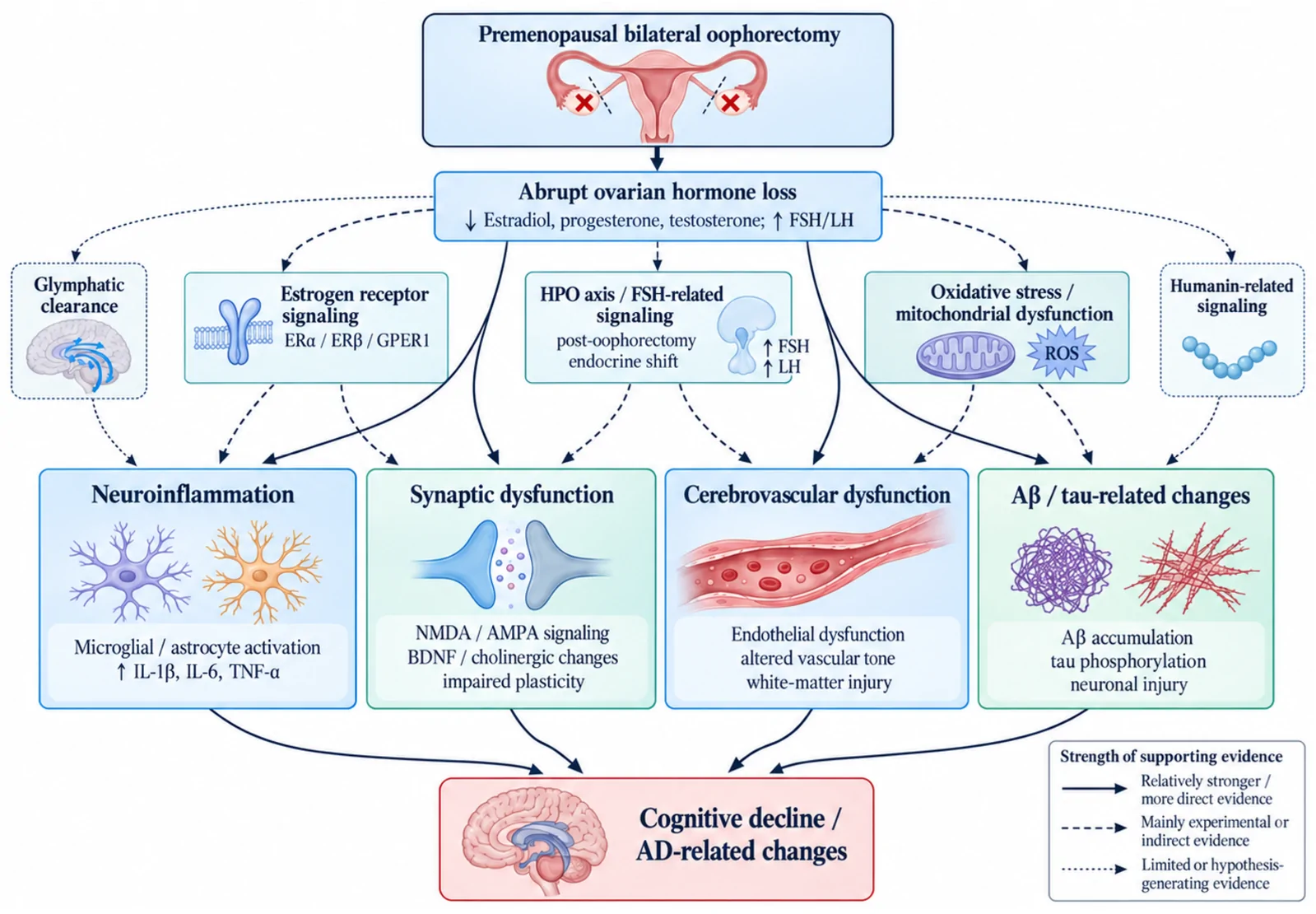
Potential biological pathways linking ovarian hormone loss to Alzheimer disease susceptibility. Solid arrows indicate relatively stronger or more direct evidence, dashed arrows indicate mainly experimental or indirect evidence, and dotted arrows indicate limited or hypothesis-generating evidence.

AD is neuropathologically characterized mainly by extracellular deposits of amyloid-β (Aβ) and intracellular neurofibrillary tangles formed by abnormal tau protein. These changes are important features of the disease, but they do not occur alone. According to the amyloid cascade hypothesis, accumulation of Aβ may contribute to a series of later changes, including abnormal tau phosphorylation, impaired synaptic function, inflammatory responses, and neuronal injury [32]. At the same time, current understanding of AD has moved beyond a single amyloid-centered pathway; neuroinflammation, cerebrovascular dysfunction, metabolic changes, and interactions among these processes are also involved in disease development [33]. Therefore, the possible effect of ovarian hormone loss on AD is more likely to involve several connected pathways rather than one isolated mechanism. However, most evidence linking these pathways specifically to oophorectomy comes from animal models or indirect human studies, so the mechanisms discussed below should be considered possible explanations rather than established causal pathways. The major biological pathways proposed to link ovarian hormone loss after oophorectomy with AD-related changes are summarized in Figure 1.

Amyloid precursor protein (APP) is involved in normal neuronal and synaptic function, while it also serves as the precursor of Aβ [34]. Under pathological conditions, sequential cleavage of APP by β-secretase (BACE1) and γ-secretase generates Aβ peptides, and Aβ42 is more likely to aggregate than shorter forms [32]. Changes in Aβ production and clearance can interact with inflammatory responses in the brain; activated microglia and other immune cells may participate in Aβ removal under some conditions, but persistent or excessive inflammatory activation can also damage neurons and synapses. In AD, prolonged neuroinflammation is commonly accompanied by changes in inflammatory mediators such as IL-1β, IL-6, and TNF-α, together with increased oxidative stress, and these changes may further worsen neuronal dysfunction [33]. Ovarian hormones may influence both inflammatory signaling and Aβ-related processes, which provides a possible biological link between hormone loss and AD-related changes, although direct evidence after oophorectomy in women remains limited.

Cerebrovascular changes are another important part of AD-related pathology and may interact with both amyloid accumulation and neuronal injury. Brain arterial aging, characterized by elastin loss, internal elastic lamina (IEL) gaps, and medial amyloid deposition, has been associated with AD neuropathology in autopsy studies (OR = 1.022, *p* = 0.002) [35]. Vascular aging may reduce cerebral perfusion and interfere with the removal of metabolic waste from brain tissue; impaired perivascular and glymphatic clearance may then contribute to the accumulation of Aβ and other harmful products [33,36]. Vascular injury and neurodegenerative changes may influence each other, while estrogen can also affect endothelial function and vascular tone; therefore, early ovarian hormone loss may be related to this pathway, but the link remains mainly indirect.

### 4.1. Potential Mechanisms Linking Oophorectomy to Neurodegeneration

The loss of ovarian hormones after oophorectomy may influence several processes that are also involved in AD, including neuroinflammation, synaptic dysfunction, and cerebrovascular changes. These pathways are closely related to each other and are unlikely to act independently. Neuroinflammation is one possible connection; studies using ovariectomized (OVX) animals have reported increased activation of microglia and astrocytes in the hippocampus, together with changes in pro-inflammatory cytokines such as IL-1β and TNF-α [37]. Because estradiol can influence glial activity and inflammatory signaling, its rapid loss after ovariectomy may shift the local brain environment toward a more inflammatory state, although the extent of this effect varies among experimental models. APOE ε4 may provide an additional source of vulnerability because this genotype is associated with altered microglial responses, lipid handling, and Aβ clearance; however, evidence that APOE ε4 specifically amplifies oophorectomy-induced neuroinflammation remains limited [38]. Most of these findings come from experimental models or general AD research, and direct evidence in women after surgical menopause is still lacking.

Synaptic dysfunction provides another possible pathway linking ovarian hormone loss with cognitive changes. Ovarian hormones, particularly estradiol, can influence synaptic plasticity through several processes, including NMDA and AMPA receptor signaling, dendritic spine formation, cholinergic function, and neurotrophic pathways such as BDNF. Experimental studies in OVX rodents have reported changes in hippocampal cholinergic activity; for example, a reduction in high-affinity choline uptake (HACU) has been observed after ovariectomy, while estradiol treatment improved this measure in some experimental conditions [31]. Estradiol may also influence long-term potentiation (LTP) in the hippocampal CA1 region through changes in glutamatergic receptor signaling, including NMDA and AMPA receptors [39]. Together, these findings suggest that loss of ovarian hormones can alter several systems involved in learning and memory, although the magnitude of these effects depends on the experimental model, hormone dose, and timing of replacement.

Mitochondrial dysfunction and altered astrocyte support may also contribute to neuronal vulnerability after hormone loss, but current evidence is mainly experimental. Humanin and other mitochondrial-derived signals have been linked to synaptic protection in laboratory studies; however, there is no direct evidence that changes in these signals after oophorectomy contribute to AD in humans [40].

Cerebrovascular function may provide another connection between early ovarian hormone loss and later cognitive health. Estrogen acts on vascular endothelium and can influence nitric oxide-dependent vasodilation; accordingly, experimental studies in ovariectomized animals have reported changes in cerebral vascular function, while estradiol treatment can improve some vascular measures under selected experimental conditions [41]. However, the vascular response to estrogen is influenced by age and the condition of the vessel, and the benefit observed in younger experimental animals should not be assumed to occur equally at all ages.

Human studies also suggest that early bilateral oophorectomy may be associated with later changes in brain structure, including lower white matter integrity in some later-life imaging studies [18]; these findings may be relevant because cerebrovascular and white matter injury can contribute to cognitive decline. However, direct evidence that oophorectomy causes long-term changes in cerebral perfusion remains limited. Vascular dysfunction could also interfere with Aβ clearance through perivascular pathways. The glymphatic system participates in the movement and removal of interstitial solutes and depends partly on vascular and astroglial function, including aquaporin-4 (AQP4)-related pathways [36]. However, direct evidence showing that oophorectomy causes glymphatic dysfunction in women is currently lacking, so this pathway should be regarded as a biologically plausible mechanism rather than an established consequence of surgery.

### 4.2. Estrogen Signaling and Context-Dependent Neuroprotection

Estrogen can influence brain function through several receptor pathways. Classical genomic signaling is mainly mediated by the nuclear estrogen receptors ERα and ERβ, while membrane-associated receptors, including G protein-coupled estrogen receptor 1 (GPER1/GPR30), can produce more rapid cellular responses. Through these pathways, estrogen signaling may affect synaptic activity, inflammatory responses, mitochondrial function, and cellular stress, all of which are relevant to brain aging and AD-related changes [24]. ERα and ERβ are expressed in brain regions involved in learning and memory, including the hippocampus and cortex, although their distribution and function differ across cell types and brain regions. Experimental studies suggest that estrogen receptor signaling can influence Aβ-related processes, oxidative stress, and synaptic plasticity; ERα has also been linked to changes in glutamatergic signaling and dendritic spine formation, while GPER1 can activate intracellular kinase pathways involved in neuronal survival [24,39].

Experimental models provide further evidence that ovarian hormones can influence AD-related changes, although the findings vary among models and should not be directly translated into human dementia prevention. In animal models of AD, treatment with ovarian hormones or estradiol has been associated with improvements in memory-related performance, cholinergic function, neuronal survival, and glial responses [42]. In the App-NL-G-F knock-in mouse model, ERβ signaling has also been linked to sex-dependent differences in AD-like pathology, providing additional support for a role of estrogen receptor signaling in the response to amyloid-related changes [24]. Overall, these experimental findings suggest that estrogen may influence several processes related to neuronal function and AD pathology, but the effect varies with the animal model, age, hormone exposure, and treatment timing, so the same benefit cannot be assumed in women after surgical menopause.

However, the effects of estrogen on the brain are context-dependent and may differ according to age, the duration of hormone deprivation, underlying brain and vascular health, and the timing and type of hormone exposure. Experimental findings obtained in young ovariectomized animals therefore cannot be assumed to apply directly to women many years after menopause. This difference between experimental models and clinical populations is important, because rodent ovariectomy usually occurs under controlled conditions and over a much shorter time scale than human reproductive aging or surgical menopause. This difference is particularly important when considering menopausal hormone therapy (MHT), because possible benefits and risks are influenced by the age at treatment initiation, the interval since menopause, the estrogen formulation and route of administration, and whether a progestogen is required. The effects of estrogen outside the brain also need to be considered when hormone therapy is discussed. MHT may have different risk profiles depending on the regimen and the clinical characteristics of the woman, and concerns such as breast cancer and thromboembolic risk are important when treatment decisions are made [43]. For this reason, interest has also developed in approaches that may provide more selective estrogen-related signaling, including selective estrogen receptor modulators (SERMs) and some phytoestrogens. Genistein, a soy-derived isoflavone, has shown possible neuroprotective effects in experimental AD models [44], but current clinical evidence is not sufficient to consider phytoestrogens an established strategy for preventing cognitive decline or AD after oophorectomy. These approaches remain mainly experimental in the context of post-oophorectomy cognitive health and should not be considered alternatives to standard management when hormone therapy is clinically indicated. The principal molecular and cellular pathways discussed above are summarized in Table 2.

## 5. Treatment and Management Considerations

### 5.1. Pharmacological Treatment of Cognitive Impairment and AD

There is currently no pharmacological treatment developed specifically for women who develop cognitive impairment or AD after oophorectomy; therefore, treatment generally follows the same principles used for other patients with AD. Cholinesterase inhibitors, including donepezil, rivastigmine, and galantamine, are commonly used as symptomatic treatments and act mainly by increasing the availability of acetylcholine at neuronal synapses. Their use is based on evidence from the general AD population rather than from women with previous oophorectomy. The ASCOMALVA trial reported that the combination of donepezil and choline alphoscerate was associated with greater improvement in some behavioral measures than donepezil alone in patients with AD [45]; however, this study did not specifically include women after surgical menopause, and it should therefore be considered as general AD treatment evidence rather than evidence for an oophorectomy-specific approach. For this reason, the findings are useful mainly as background information on symptomatic AD treatment and should not be interpreted as evidence that women after oophorectomy require a different treatment strategy.

Memantine, an uncompetitive NMDA receptor antagonist, is another symptomatic treatment used in AD, particularly in moderate-to-severe disease; it reduces excessive NMDA receptor activity while allowing normal glutamatergic signaling to continue. Some experimental and clinical studies have also described effects of memantine on immune signaling, including changes in Kv1.3 potassium-channel activity and T-cell responses [46], but these findings remain indirect in the context of surgical menopause. In women with previous oophorectomy, memantine should therefore be used according to the usual clinical indications for AD rather than because of the surgical history itself.

Statins have also been studied in relation to cognition and ovarian hormone loss, but the evidence is much less direct. In ovariectomized rats, rosuvastatin has been associated with improvement in spatial learning and memory together with changes in oxidative stress and inflammatory measures [47]. These findings are mainly experimental and do not support the use of statins as a treatment for post-oophorectomy cognitive decline. In clinical practice, statins should still be used for established cardiovascular or lipid-lowering indications, while any specific cognitive benefit after oophorectomy remains uncertain.

More recently, disease-modifying anti-amyloid therapies have become available for selected patients with early AD. These treatments are discussed later in this review because they are relevant only when AD has been clinically and biomarker confirmed; at present, there is no evidence that a history of oophorectomy changes the usual indications for treatment.

### 5.2. Non-Pharmacological Approaches

Non-pharmacological measures may be useful as part of a broader approach to maintaining cognitive and general health after oophorectomy, although no cognitive-training program has been specifically proven to prevent dementia in this population. Cognitive training has been studied in several groups with cognitive impairment, and some studies have reported improvements in attention, memory, or other cognitive measures; however, the results depend on the population, type of training, and duration of intervention. Because most cognitive-training studies were not performed in women after oophorectomy, their relevance to surgical menopause remains uncertain. These findings suggest that cognitive stimulation may support cognitive function in selected individuals, but the available evidence does not show that it can specifically prevent AD after premenopausal oophorectomy.

Regular physical activity is one of the more practical non-pharmacological measures for maintaining health during aging, and it may be particularly relevant after early surgical menopause because cardiovascular, metabolic, bone, and cognitive health are closely related. Experimental studies have suggested that exercise can influence brain-derived neurotrophic pathways and inflammatory or oxidative processes; for example, exercise-related FNDC5/irisin signaling has been associated with improved cognitive performance in aged animal models [48]. Human evidence also generally supports physical activity as part of dementia-risk reduction, although the amount, intensity, and type of exercise that would be most useful specifically after oophorectomy have not been established. Therefore, exercise should be considered a general health and risk-reduction measure rather than a proven treatment for oophorectomy-related cognitive decline.

Diet is another part of general long-term health management and may influence cognitive risk mainly through metabolic, vascular, and inflammatory pathways. A Mediterranean-style dietary pattern, which usually contains more vegetables, fruits, whole grains, fish, and sources of unsaturated fatty acids, has been associated with better cognitive outcomes and a lower risk of AD in observational research [49]. Other dietary strategies and gut–brain-related interventions remain under investigation, but current evidence is too indirect to support their use specifically for preventing cognitive decline after oophorectomy.

Overall, diet, exercise, cognitive activity, smoking avoidance, and control of vascular and metabolic risk factors are more appropriately viewed as parts of a broader long-term health strategy rather than as established treatments for hormone-loss-related AD. For women who undergo oophorectomy before natural menopause, these measures may be especially relevant because cardiovascular and metabolic factors may also influence later cognitive health, although direct evidence for an oophorectomy-specific benefit is still lacking.

### 5.3. Hormone Replacement Therapy and the Critical Window Hypothesis

Hormone replacement therapy (HRT) has an established role in the management of premature or early surgical menopause when there is no contraindication, mainly because the abrupt loss of ovarian hormones can cause vasomotor symptoms and may also affect bone, cardiovascular, and genitourinary health. Its possible effect on long-term cognitive outcomes, however, remains less certain. A systematic review of 13 observational studies found that short-term HRT use (<5 years) initiated after RRSO was associated with improvement in quality of life and did not show a clear increase in breast cancer risk during the available follow-up [50]. These findings support the use of HRT for management of surgical menopause, but they should not be interpreted as direct evidence that HRT prevents cognitive decline or AD.

Evidence from observational studies after premenopausal oophorectomy has suggested that the timing and duration of hormone therapy may be important, although the findings are not fully consistent. In the Mayo Clinic cohort, some earlier analyses suggested that estrogen treatment continued to around the usual age of natural menopause might reduce part of the excess long-term cognitive risk associated with early oophorectomy; however, later analyses did not show a clear protective association in all subgroups [12,30]. These differences may reflect variation in age at surgery, treatment duration, indication for surgery, and other baseline characteristics.

**Figure 2:**
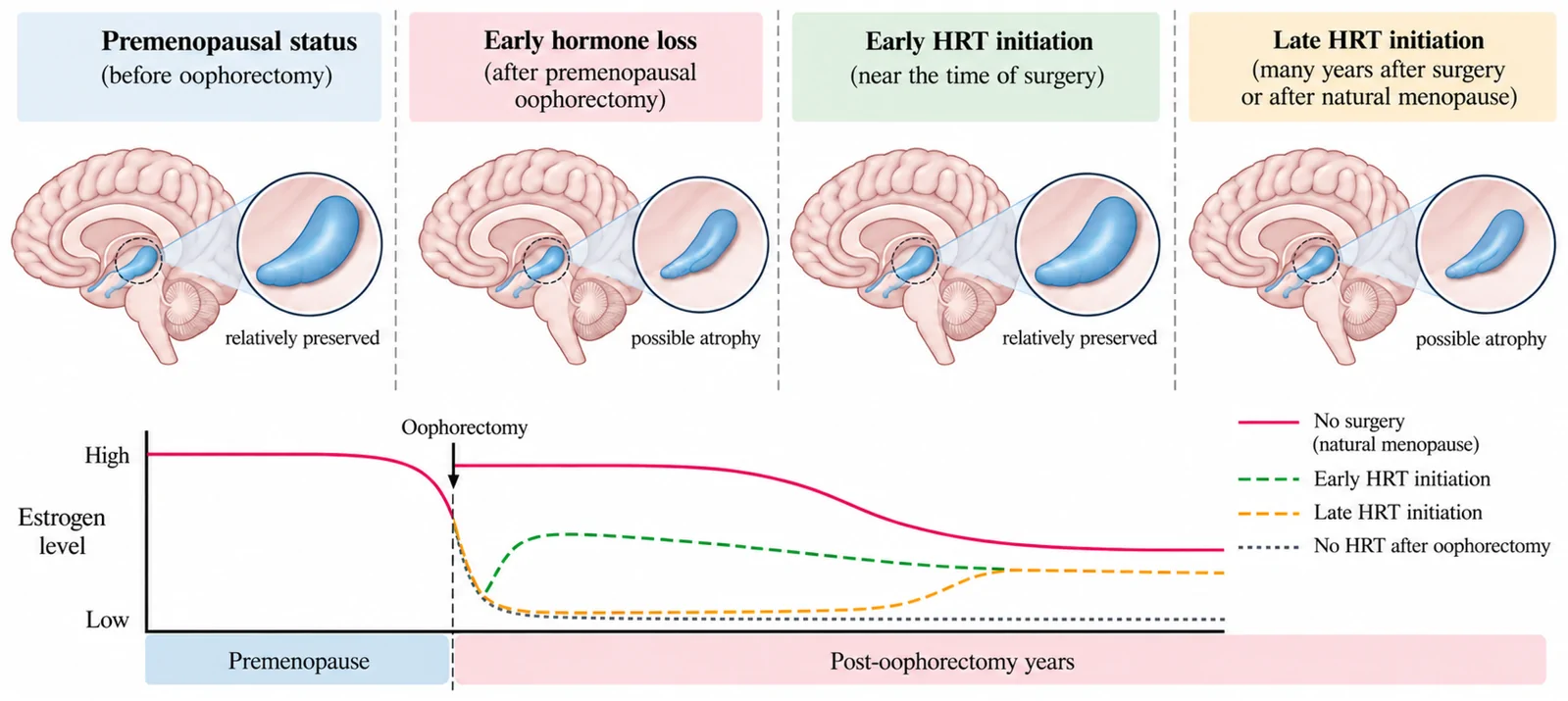
Conceptual framework of hormone replacement therapy timing and the critical-window hypothesis after premenopausal bilateral oophorectomy. The schematic shows possible differences in hippocampal preservation with early versus late HRT initiation; these changes are illustrative and do not represent established causal effects.

The “window of opportunity” hypothesis proposes that the effect of HRT may depend partly on when treatment is started relative to menopause; treatment initiated closer to the onset of hormone loss may produce different effects from treatment begun many years later [29]. This concept is biologically plausible, but clinical studies have produced mixed results, and most trials have been performed in women with natural rather than surgical menopause. Observational neuroimaging studies have also reported differences in brain structure according to the timing of hormone exposure; for example, one study found that women who started HRT at a younger age had larger hippocampal volumes than women who initiated treatment later [51]. Although this finding is consistent with a possible timing effect, it does not establish that earlier HRT prevents later dementia. The proposed relationship between the timing of ovarian hormone loss, HRT initiation, and later cognitive outcomes is illustrated in Figure 2. The route of estrogen administration is an important part of treatment selection, but the choice should be based on the woman’s age, symptoms, vascular risk, medical history, and whether the uterus is present. Transdermal estradiol avoids first-pass hepatic metabolism and may be preferred in some women, particularly when thrombotic or metabolic risk is a concern; however, there is no clear evidence that the transdermal route provides greater protection against cognitive decline or AD than oral estrogen [43]. The formulation and dose should therefore be selected according to general menopausal treatment principles rather than an assumed cognitive benefit. For women with an intact uterus, adequate endometrial protection with progesterone or another progestogen is required when systemic estrogen is used. Micronized progesterone is often considered because of differences in metabolic and tolerability profiles compared with some synthetic progestins, but evidence for a specific cognitive advantage remains limited [43]. Women who have undergone hysterectomy generally do not require a progestogen for endometrial protection.

Despite the theoretical rationale and some supportive observational findings, high-quality randomized evidence addressing dementia prevention specifically in women after premenopausal oophorectomy remains limited. This is an important limitation because most available hormone-therapy trials were not designed for this population. The Women’s Health Initiative Memory Study (WHIMS) included women aged 65 years and older rather than women who had recently undergone premenopausal oophorectomy. In the estrogen-plus-progestin arm, hormone therapy was associated with a higher risk of probable dementia in older postmenopausal women [52], while the estrogen-alone arm in women with previous hysterectomy also did not show a preventive cognitive benefit [53]. These findings are important for older women, but they should not be directly applied to younger women who begin HRT soon after surgical menopause. The Kronos Early Estrogen Prevention Study (KEEPS) examined hormone therapy in women who were much closer to the onset of natural menopause and found no clear adverse effect on cognition over the study period; some differences in mood-related outcomes were also reported [54]. Longer-term follow-up from KEEPS has also not shown a clear persistent cognitive benefit or harm from the earlier treatment period [55]. Importantly, KEEPS included women after natural menopause rather than women with surgical menopause, so its findings cannot be directly transferred to women after premenopausal oophorectomy.

The Early versus Late Intervention Trial with Estradiol (ELITE) directly examined whether the timing of estrogen initiation influenced cognitive outcomes by comparing women who were closer to menopause with women who were many years beyond menopause. The trial did not show a clear cognitive advantage of earlier estrogen initiation, which suggests that the timing hypothesis may not apply equally to all outcomes [56]. Large registry studies have also produced mixed findings. Some observational data from Denmark have reported an association between menopausal hormone therapy and a higher rate of dementia, including among women who started treatment at a relatively younger age [57]; however, these studies remain subject to confounding by indication, treatment pattern, duration of use, and differences between women who receive or do not receive hormone therapy.

Taken together, WHIMS, KEEPS, ELITE, observational cohorts, and registry studies do not provide a simple answer regarding HRT and later cognition. Age at treatment, type of menopause, time since hormone loss, formulation, duration of treatment, and baseline vascular risk may all influence the observed results; however, current evidence does not show that HRT should be prescribed specifically for prevention of AD. In women with premature or early surgical menopause, the main indication for HRT remains the management of consequences related to early hormone loss, while any possible long-term cognitive effect should be considered uncertain.

### 5.4. Phytoestrogens and Other Alternative Approaches

Phytoestrogens have attracted attention because some compounds can interact with estrogen-related signaling and may therefore provide biological effects that partly overlap with endogenous estrogen. However, they should not be considered a clearly safer substitute for conventional hormone therapy, especially when long-term cognitive outcomes are the main concern. Experimental studies have reported several possible effects of phytoestrogens after ovarian hormone loss; for example, flaxseed-derived compounds have been studied mainly for metabolic, hormonal, and bone-related changes in ovariectomized animals [58], while genistein has shown possible effects on Aβ-related processes and cognitive performance in experimental AD models [44]. These findings are of biological interest, but most of the evidence remains preclinical and does not establish that phytoestrogens can prevent cognitive decline or AD in women after oophorectomy.

Clinical evidence is much more limited, and studies of alternative treatments in postmenopausal women have often focused on menopausal symptoms rather than cognition. Therefore, positive findings for symptoms or other organ systems should not be interpreted as evidence of neuroprotection. At present, phytoestrogens and similar approaches should mainly be regarded as research topics. They should not replace clinically indicated HRT after premature surgical menopause, and current evidence does not support their use for AD prevention.

### 5.5. Clinical Implications and Risk-Reduction Strategies

Clinical management before and after oophorectomy should consider both the reason for surgery and the possible long-term effects of premature ovarian hormone loss. When surgery is performed for benign gynecological disease, ovarian conservation may be considered when it is oncologically and clinically appropriate, especially in younger premenopausal women. The decision should still depend on age, ovarian pathology, family history, genetic risk, and the expected benefits and risks of preserving ovarian function.

For women undergoing hysterectomy for benign disease, opportunistic salpingectomy has been increasingly used as a possible strategy to reduce future tubo-ovarian cancer risk while preserving the ovaries [59]. This approach may avoid immediate surgical menopause because the ovaries are left in place, although the long-term effects on ovarian function should still be considered. In women with BRCA1/2 pathogenic variants, risk-reducing salpingo-oophorectomy remains the standard preventive approach, but salpingectomy with delayed oophorectomy is being studied as an alternative strategy for women who wish to postpone premature menopause. Studies such as TUBA and WISP have examined this approach, mainly with attention to quality of life, menopausal symptoms, and cancer-risk management [60,61]; long-term cognitive outcomes are not yet established.

After premenopausal oophorectomy, follow-up should not focus only on menopausal symptoms. Bone health, cardiovascular risk, metabolic factors, sleep, mood, and cognitive complaints may also need attention, especially in women who undergo surgery at a younger age. Formal cognitive testing is not routinely required for all women after surgery, but baseline or follow-up assessment may be considered when persistent cognitive symptoms are reported or when several additional risk factors are present. These may include APOE ε4 carrier status, cardiovascular disease, depression, low educational attainment, or a strong family history of dementia.

Hormone therapy should be considered according to established indications for premature or early surgical menopause rather than for the specific purpose of preventing AD. When HRT is used, the timing of initiation, route, dose, treatment duration, hysterectomy status, and individual vascular or cancer risk should be considered [8,43]. For women with an intact uterus, appropriate endometrial protection is required when systemic estrogen is prescribed [43].

Overall, the main clinical aim is not to treat oophorectomy as a direct cause of AD, but to reduce avoidable loss of ovarian function when appropriate, manage the consequences of early surgical menopause, and identify women who may benefit from closer long-term follow-up.

## 6. Emerging Approaches and Future Research Directions

### 6.1. Emerging Therapeutic and Experimental Approaches

Treatment of AD has changed in recent years with the development of disease-modifying anti-amyloid therapies. These agents are relevant to women with previous oophorectomy only if they later develop biomarker-confirmed early AD, and there is currently no evidence that a history of oophorectomy changes their treatment response. Anti-amyloid antibodies can reduce cerebral amyloid burden and may slow clinical decline in selected patients, but the benefit is modest and treatment requires careful monitoring because amyloid-related imaging abnormalities (ARIA), including edema and hemorrhagic changes, may occur [62]. Sex-related differences in treatment response have also received attention. Some subgroup analyses of lecanemab trials have suggested that the magnitude of clinical benefit may differ between women and men, but these analyses were not designed to determine whether sex hormones or previous oophorectomy explain the difference [62]. Current evidence also does not show that surgical menopause or HRT use clearly changes the risk of ARIA. These questions remain uncertain and require more direct study. Therefore, these drugs should be viewed as treatments for established early AD rather than as preventive therapies for women undergoing oophorectomy.

Gene-editing approaches, including CRISPR-based strategies, are also being explored in experimental AD research, particularly for genes such as APOE. However, these methods remain at an early stage and are far from routine clinical use; at present, they are more relevant as research tools for understanding genetic risk than as realistic treatment options for post-oophorectomy cognitive decline [63]. Other emerging approaches are mainly focused on improving drug delivery or modifying the cellular environment. Nanomedicine is one example because the blood–brain barrier limits the delivery of many drugs to the central nervous system. Nanoparticle-based systems are being developed to improve drug transport, target Aβ or other pathological processes, and reduce off-target exposure; however, most evidence remains preclinical, and clinical benefit in AD has not yet been clearly established [64]. For women after oophorectomy, brain-targeted delivery of estrogen or other neuroactive compounds remains mainly a theoretical possibility, and there is currently no clinical evidence supporting this strategy.

Extracellular vesicles (EVs), including those derived from mesenchymal stem cells, have also been studied as possible carriers of proteins, lipids, and regulatory molecules that may influence inflammation or neuronal repair. Some experimental AD studies have reported favorable changes in inflammatory and neuronal outcomes, but this field remains at an early stage and there is no evidence that EV-based treatment is effective specifically after surgical menopause [65].

In addition to these potential therapeutic approaches, preclinical models may help clarify the mechanisms linking ovarian hormone loss with AD-related changes. Induced pluripotent stem cells (iPSCs) allow human neuronal and glial cells to be generated from individuals with different genetic backgrounds, including different APOE genotypes; these models can be used to examine how hormone exposure, hormone withdrawal, and genetic susceptibility affect Aβ- and tau-related changes, inflammatory responses, and cellular stress [66]. Three-dimensional brain organoids may provide an additional model because they allow several neural cell types to interact in a more complex environment than conventional cell culture [67]. For this review, the main value of these models is not that they already provide a treatment, but that they may help separate the effects of ovarian hormone loss from the effects of age, genetic risk, and vascular factors. Future models comparing estrogen exposure, withdrawal, and replacement under different APOE backgrounds may provide more specific mechanistic evidence for the oophorectomy–AD relationship, although these findings will still require confirmation in human studies.

### 6.2. Future Research Priorities

For women undergoing premenopausal oophorectomy, several questions remain more important than the development of highly experimental therapies. First, larger prospective cohorts are needed, with detailed recording of the indication for surgery, age and menopausal stage at oophorectomy, unilateral or bilateral surgery, hysterectomy status, and hormone therapy exposure, including type, dose, route, duration, and timing of initiation. Long-term cognitive outcomes should ideally be combined with repeated neuropsychological assessment, brain imaging, and AD-related biomarkers. Second, future studies should examine whether the effects of hormone therapy differ according to the timing of treatment, age at surgery, duration of hormone deprivation, and baseline vascular or genetic risk. Randomized studies may be difficult because very long follow-up would be required, but carefully designed prospective or controlled studies with cognitive, imaging, and biomarker outcomes could still provide useful evidence. Third, the interaction between APOEε4, cardiovascular risk factors, lifestyle factors, and surgical menopause needs further study, because these factors may partly explain why cognitive outcomes differ among women with similar surgical histories. Such studies may also help identify women who are more vulnerable after early loss of ovarian function, rather than treating all women with the same surgical history as having the same level of risk.

Alongside these clinical questions, mechanistic studies may help explain why the effects of ovarian hormone loss differ among individuals. Experimental work on brain-selective estrogen signaling, iPSC models, gene editing, nanomedicine, and related technologies may help explain the underlying mechanisms, but better human evidence remains the more immediate need.

Future research should also pay more attention to populations that are currently underrepresented. Long-term cognitive data are very limited in transgender men who undergo oophorectomy as part of gender-affirming care, and available studies mainly describe endocrine, reproductive, or ovarian changes rather than dementia or cognitive outcomes [6]. Therefore, this population should currently be regarded as an important evidence gap rather than as a group with an established increase in AD risk. More studies are also needed in women from different ethnic and geographic backgrounds, because much of the current evidence still comes from a limited number of Western cohorts.

## 7. Discussion

Current evidence suggests that premenopausal bilateral oophorectomy may be associated with later cognitive impairment, particularly when surgery is performed at a younger age, but the findings are not fully consistent. Different studies have assessed all-cause dementia, mild cognitive impairment, subjective cognitive symptoms, or imaging changes, and these outcomes should not be considered equivalent. The observed associations may also be influenced by the indication for surgery, hysterectomy status, cardiovascular and psychological health, socioeconomic factors, and HRT use. Healthy-user bias, survivor bias, reverse causation, and residual confounding may therefore partly explain the differences between studies.

Another important limitation is that the definition of surgical menopause is not completely consistent across studies, and some cohorts include women with hysterectomy, unilateral oophorectomy, or different indications for surgery. In addition, much of the mechanistic evidence comes from ovariectomized animal models or indirect human studies, which limits direct translation to women after premenopausal oophorectomy. A strength of the current literature is that epidemiological, neuroimaging, and experimental findings provide several complementary lines of evidence; however, direct causal evidence remains limited.

A strength of this review is the integration of epidemiological, neuroimaging, hormonal, and experimental evidence while distinguishing clinical AD from related cognitive and imaging outcomes. As a narrative review, however, it did not include a formal systematic risk-of-bias assessment or quantitative evidence synthesis, which should be considered when interpreting the conclusions.

## 8. Conclusions

Premenopausal bilateral oophorectomy may be associated with later cognitive impairment, especially when surgery occurs at a younger age, but current evidence does not establish a direct causal relationship with AD. HRT remains important for the management of premature or early surgical menopause when clinically indicated, although its role in preventing AD is uncertain. Future prospective studies with detailed information on surgical indication, hormone exposure, vascular and genetic risk, and long-term cognitive outcomes are needed.

## Figures and Tables

**Table 1:** Summary of clinical and epidemiological evidence linking oophorectomy or surgical menopause to cognitive and brain outcomes.

Study	Population/Exposure and Follow-Up	Outcome Ascertainment	Main Finding	Adjustment/HRT Definition	Evidence Appraisal
Rocca et al. [12]	Mayo cohort; premenopausal oophorectomy; long-term follow-up.	Medical records and follow-up for cognitive impairment/dementia.	Higher later risk; stronger with younger surgical age.	Age at surgery and estrogen use considered; estrogen to ~age 50 examined.	Long follow-up; observational; residual confounding possible.
Georgakis et al. [10]	Meta-analysis of 11 studies; early surgical menopause.	Dementia and cognitive outcomes as defined in each study.	Age ≤ 45 y: HR 1.70 (95% CI 1.07–2.69).	Adjusted estimates from included studies; HRT definitions varied.	Higher-level evidence; marked heterogeneity.
Rocca et al. [13]	Mayo cohort; PBO < 46 y; ~30 y interval to cognitive assessment.	Neuropsychological testing and clinical assessment for MCI.	MCI aOR 2.21 (95% CI 1.41–3.45); stronger for benign indications.	Multivariable adjustment; estrogen use assessed.	Long-term cohort; MCI, not AD; residual confounding possible.
Uldbjerg et al. [14]	Danish Nurse Cohort; 24,851 women; registry follow-up.	Incident dementia from national health registries.	Bilateral oophorectomy: aRR 1.18 (0.89–1.56), not significant.	Adjusted regression; hysterectomy and HT tested as modifiers.	Large prospective cohort; limited exposed dementia cases.
Calvo et al. [16]	Early BSO vs. comparison groups; ~5 y after surgery.	SCD, cognitive testing, and structural MRI.	More SCD and lower gray-matter volume in estrogen-sensitive regions.	Estradiol therapy and verbal IQ examined.	Multimodal assessment; SCD/imaging are not clinical AD.
Kantarci et al. [17]	PBO < 46 y, 46–49 y, and referents; later-life imaging.	Aβ PET, tau PET, and MRI cortical measures.	Higher Aβ burden, thinner entorhinal cortex, and greater AD-related cortical atrophy after early PBO.	Age and APOE ε4 adjusted; HT through age 50 examined.	Direct AD biomarker imaging; cross-sectional imaging limits causality.
Mielke et al. [18]	PBO < 40, 40–45, 46–49 y vs. referents; later-life MRI.	DTI fractional anisotropy and mean diffusivity.	PBO < 40 y: lower FA and higher MD in several regions.	Age/APOE adjusted; further cardiovascular and ERT adjustment.	Objective imaging; surrogate outcome only.
Maki et al. [19] (WHAM)	BRCA1/2 carriers undergoing RRSO vs. controls; 24 mo.	Serial standardized neuropsychological testing.	Most domains similar; smaller improvement in verbal learning after RRSO.	Baseline factors adjusted; HT type, timing, and duration modeled.	Prospective controlled study; short follow-up and BRCA-specific cohort.
Chang et al. [20]	High-risk women undergoing RRSO; 6–18 mo.	FACT-Cog and wellbeing measures.	Short-term change in perceived cognition; other domains largely stable.	HT use included in longitudinal analyses.	Small sample; short follow-up; mainly subjective outcomes.
Calvo et al. [21]	UK Biobank; early BO vs. spontaneous menopause; *n* = 34,603.	AD identified by ICD-9/ICD-10 codes.	Early BO associated with higher odds of coded AD.	Age, education, menopause age, HT, APOE4, BMI, cancer, smoking adjusted.	Large cohort; ICD-coded AD and residual confounding are limitations.
Barth et al. [28]	UK Biobank MHT users; hysterectomy ± BSO.	MRI brain-age gap, hippocampal volume, and WMH.	Small difference in gray-matter brain-age gap.	Age, education, BMI, lifestyle, menopause status adjusted; MHT data available.	Large imaging cohort; secondary observational subgroup analysis.

**Table 2:** Proposed biological pathways linking ovarian hormone loss after oophorectomy to AD-related changes.

Pathway	Key Components	Possible Effect After Hormone Loss	Evidence	Main Limitation
Neuroinflammation	Microglia; astrocytes; IL-1β; IL-6; TNF-α	↑ Inflammatory signaling; glial activation	Human AD data + OVX animal studies	Direct post-oophorectomy human evidence limited
Synaptic dysfunction	NMDA; AMPA; BDNF; cholinergic signaling; LTP	Reduced synaptic plasticity; impaired learning/memory pathways	Mainly animal/experimental	Effects vary by model, hormone dose, and timing
Aβ/tau-related changes	APP; BACE1; Aβ; tau; estrogen receptors	Altered Aβ processing; tau phosphorylation; neuronal injury	General AD biology + experimental models	Direct causal link after oophorectomy not established
Cerebrovascular dysfunction	Endothelium; nitric oxide; vascular tone; white matter	Reduced vascular support; possible white-matter injury	Human imaging + animal vascular studies	Cerebral perfusion effects after oophorectomy remain unclear
Estrogen receptor signaling	ERα; ERβ; GPER1	Changes in genomic/membrane signaling; effects on synapses and inflammation	Experimental + mechanistic studies	Context-dependent; no consistent clinical neuroprotection
HPO axis/FSH signaling	FSH; LH; HPO axis	↑ Gonadotropins; possible effects on Aβ/tau pathways	Mainly animal studies	Independent effect in women remains uncertain
Oxidative stress/mitochondrial dysfunction	ROS; mitochondrial signaling	↑ Cellular stress; reduced neuronal support	Mainly experimental	Human evidence after surgical menopause limited
Glymphatic clearance	AQP4; perivascular clearance	Possible reduction in waste clearance and Aβ removal	Indirect/general AD evidence	No direct evidence after oophorectomy
Humanin-related signaling	Humanin; astrocytes; mitochondrial peptides	Possible synaptic protection	Experimental only	Very limited relevance to human post-oophorectomy AD

Note: ↑ indicates an increase in the specified biological process or signaling activity.

## Data Availability

No new data were created or analyzed in this study. Data sharing is not applicable to this article.

## Abbreviations

The following abbreviations are used in this manuscript:

AD	Alzheimer’s disease
APOE	Apolipoprotein E
Aβ	Amyloid-β
ARIA	Amyloid-related imaging abnormalities
BDNF	Brain-derived neurotrophic factor
BSO	Bilateral salpingo-oophorectomy
DTI	Diffusion tensor imaging
ERα	Estrogen receptor alpha
ERβ	Estrogen receptor beta
FA	Fractional anisotropy
FSH	Follicle-stimulating hormone
GM-BAG	Gray matter brain age gap
GPER1	G protein-coupled estrogen receptor 1
HPO	Hypothalamic-pituitary-ovarian
HRT	Hormone replacement therapy
LTP	Long-term potentiation
MCI	Mild cognitive impairment
MD	Mean diffusivity
MHT	Menopausal hormone therapy
OVX	Ovariectomized
PBO	Premenopausal bilateral oophorectomy
RRSO	Risk-reducing salpingo-oophorectomy
SCD	Subjective cognitive decline
SERM	Selective estrogen receptor modulator
